# Internal translation of the connexin 43 transcript

**DOI:** 10.1186/1478-811X-12-31

**Published:** 2014-05-08

**Authors:** Clàudia Salat-Canela, Marta Sesé, Cristina Peula, Santiago Ramón y Cajal, Trond Aasen

**Affiliations:** 1 Molecular Pathology, Hospital Universitari Vall d’Hebron - Institut de Recerca (VHIR), Universitat Autònoma de Barcelona, Passeig Vall d’Hebron 119-129, Barcelona 08035, Spain

**Keywords:** Gap junction, Connexin 43, IRES, Mnk, mTOR, Cap-dependent, Internal translation

## Abstract

**Background:**

Connexin 43 (Cx43), the most widely expressed gap junction protein, is associated with a number of physiological and pathological conditions. Many functions of Cx43 have been shown to be independent of gap junction formation and only require the expression of Cx43 C-terminal fragments. Recent evidence demonstrated that naturally occurring C-terminal isoforms can be generated via internal translation.

**Findings:**

Here, we confirm that C-terminal domains of Cx43, particularly the major 20-kDa isoform, can be independently generated and regulated by internal translation of the same single *GJA1* gene transcript that encodes full-length Cx43. Through direct RNA transfection experiments, we provide evidence that internal translation is not due to a *bona fide* cap-independent IRES-mediated mechanism, as upstream ribosomal scanning or translation is required. In addition to the mTOR pathway, we show for the first time, using both inhibitors and cells from knockout mice, that the Mnk1/2 pathway regulates the translation of the main 20-kDa isoform.

**Conclusions:**

Internal translation of the Cx43 transcript occurs but is not cap-independent and requires translation upstream of the internal start codon. In addition to the PI3K/AKT/mTOR pathway, the major 20-kDa isoform is regulated by the Mnk1/2 pathway. Our results have major implications for past and future studies describing gap junction-independent functions of Cx43 in cancer and other pathological conditions. This study provides further clues to the signalling pathways that regulate internal mRNA translation, an emerging mechanism that allows for increased protein diversity and functional complexity from a single mRNA transcript.

## Findings

Gap junctional intercellular communication, which allows the exchange of small ions and messengers between cells, is crucial for tissue development and homeostasis [1,2]. Connexin 43 (Cx43)—the most widely expressed gap junction protein—is associated with a number of pathological conditions, including cardiac, skin, neurological conditions, as well as developmental disorders and cancer [2].

Numerous studies have demonstrated that Cx43 has several junction-independent functions, many of which can be attributed solely to the C-terminus. For example, the Cx43 C-terminus has been shown to mediate neuroprotection during stroke [3], to negatively modulate neuronal differentiation [4], to augment p38-mediated cell migration [5], and to regulate cytoskeletal changes in glioma cells [6]. A naturally occurring cytoplasmic C-terminal fragment of Cx43, ~20 kDa in size, has been described and characterized in cultured murine and hamster cells and after Cx43 overexpression in human HeLa cells [7]. Others have also reported Cx43 immunoreactive bands of ~20 kDa, such as in glioma cells [8] and in neonatal rat heart myofibroblasts [9]. A 20-kDa Cx43 immunoreactive band is also clearly detected in zebrafish heart 48 hours postfertilization [10], and the authors also noted an ~30-kDa band in zebrafish ovaries. Indeed, bands at 30–32 kDa have been reported in various tissues, including the rat retina, heart, and brain [11,12].

From the large amount of literature supporting gap junction-independent functions of Cx43, usually through the C-terminus alone, and because an immunoreactive band(s) of ~20 kDa in size can occur naturally, we sought to elucidate how this fragment(s) can be produced and modulated. Recently, Smyth and Shaw [13] provided excellent evidence that internal translation of the *GJA1* gene transcript produces the 20-kDa fragment “GJA1-20k” (and, to a lesser extent, other fragments between 26 and 32 kDa in size). Here, we describe our similar findings in this context, confirming the recent report as well as adding new evidence to the mechanism of translation and the mode of regulation.

### Internal translation

**Figure 1:**
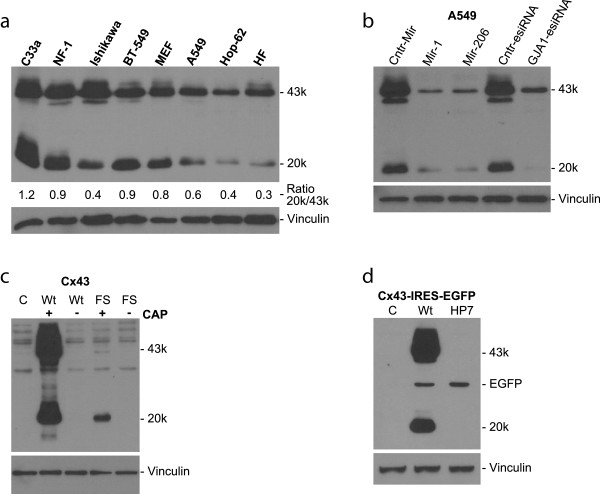
**Expression of the 20-kDa isoform is common and due to cap-dependent internal translation. (a)** Analysis of various cell lines and primary cells confirms the frequent existence of a 20-kDa isoform of Cx43 (GJA1-20k), which includes cervical cancer cells (C33a), mouse embryonic carcinoma cells (NF-1), endometrial cancer cells (Ishikawa), breast cancer cells (BT-549), mouse embryonic fibroblasts (MEFs), lung cancer cells (A549, Hop-62), and primary human fibroblasts (HFs). **(b)** Knockdown of Cx43 with esiRNA, or by microRNA-1 or −206, shows the concomitant regulation of full-length Cx43 (GJA1-43k) and the GJA1-20k isoform in lung A549 cancer cells. **(c)** Transfection of Cx43-negative HeLa cells with Cx43 RNA leads to the generation of both full-length GJA1-43k and the GJA1-20k isoform. RNA lacking the 5′ guanosine methylation (CAP), required for standard translation initiation, completely prevents both wild-type (Wt) Cx43 and internal 20-kDa fragment translation. A frameshift (FS) mutation shortly after the Cx43 AUG codon also significantly reduces internal translation, although a 20-kDa band is detected with a very long exposure time (saturating GJA1-43k). **(d)** Transfection of capped RNA containing a HP7 hairpin loop, preventing cap-dependent ribosomal scanning and translation, completely blocks both GJA1-43k and GJA1-20k expression whereas cap-independent translation of EGFP via the EMCV IRES is maintained (both anti-GFP-HRP and anti-Cx43 antibodies are included in this blot).

**Figure 2:**
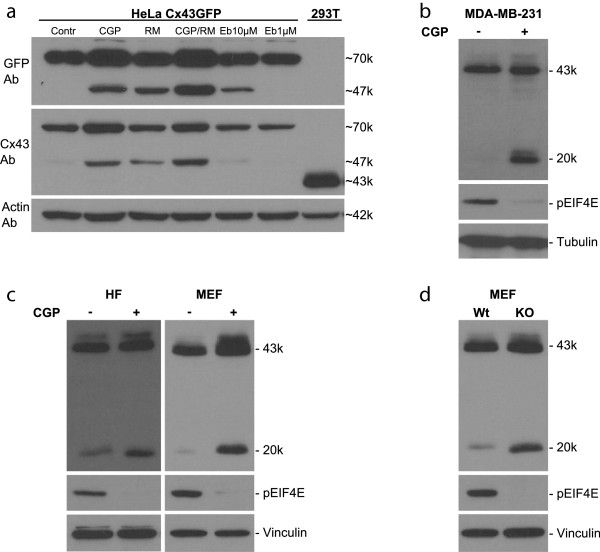
**Mnk1/2-mediated regulation of internal translation. (a)** HeLa cells stably expressing Cx43-GFP (70 kDa) show an induction of GJA1-20k (20 kDa + 27 kDa GFP = 47 kDa) upon mTOR pathway inhibition with rapamycin (RM). Inhibition of Mnk1/2 with CGP 57380 (CGP) also induces internal translation and the effect is additive upon combining CGP and RM. Inhibition of the upstream EGFR pathway with erlotinib (Eb) modestly induces internal translation in HeLa cells. **(b)** CGP also induces 20-kDa fragment expression in MDA-MB-231 cells. **(c)** The Mnk1/2 inhibitor CGP efficiently induces GJA1-20k expression levels in primary human fibroblasts (HFs) and mouse embryonic fibroblasts (MEFs). **(d)** Mnk1/2 knockout mouse embryonic fibroblasts (MEFs) have constitutively significantly higher levels of the GJA1-20k isoform than wild-type cells. The lack of S209-phosphorylated eIF4E confirms the efficient inhibition of Mnk1/2 with both CGP-treated cells and in Mnk1/2 knockout cells.

We have expanded upon the previous observations of a 20-kDa band derived from the Cx43 gene *GJA1* and corresponding to the Cx43 C-terminus (GJA1-20k) [7,13]. With antibodies targeting the last 20 amino acids of the Cx43 C-terminal tail (see Additional file 1: Methods and Additional file 2: Figure S1), we detect GJA1-20k in a wide variety of cell lines derived from different cancers including cervical cancer (C33a), lung cancer (A549 and Hop-62), breast cancer (BT-549 and MDA-MB-231), endometrial adenocarcinoma (Ishikawa), and mouse embryonic carcinoma (NF-1) (Figures 1a and 2b). Indeed, we are able to detect GJA1-20k in any cell line expressing full-length Cx43 (GJA1-43k), although, as seen in Figure 1a, the GJA1-20k to GJA1-43k ratio varies significantly. Moreover, we easily detect GJA1-20k in primary cells, including mouse embryonic fibroblasts, human mesenchymal fibroblasts, and epithelial foreskin keratinocytes (Figure 1a; see also Figure 2c-d, and Additional file 3: Figure S2A).

The specificity of the 20-kDa fragment and its correlation to Cx43 has previously been confirmed using various antibodies, siRNA-mediated knockdown, and Cx43/GJA1 knockout mice [7,13]. Indeed, correlated coregulation of GJA1-43k and -20k is observed in various settings; for example, reduced levels of both isoforms occur in primary human keratinocytes subjected to calcium-induced differentiation (Additional file 3: Figure S2A). As expected, we also observed a loss of both GJA1-43k and GJA1-20k after highly specific esiRNA-mediated knockdown (endoribonuclease-prepared siRNAs) of GJA1 (Figure 1b and Additional file 3: Figure S2B). Additionally, as Cx43 has been shown to be translationally regulated by microRNAs (*in vivo*, with biological meaning), we transfected cells with microRNA-1 and −206 mimics known to target Cx43 [14-16]. We observed significant knockdown of both GJA1-43k and GJA1-20k in lung A549 cells (Figure 1b) as well as in breast BT-549 cells (Additional file 3: Figure S2B). This result suggests that any putative smaller isoforms of Cx43 are also likely to be regulated by microRNAs *in vivo*. It is worth noting, however, that, unlike esiRNA-mediated knockdown, microRNAs mimics appeared more efficient at repressing the expression of GJA1-43k than GJA1-20k. It will be interesting to study this observation further as knowledge of microRNA-mediated translational repression expands.

As full-length Cx43 stems from a single coding exon of the Cx43 gene *GJA1*, we hypothesized that generation of the 20-kDa fragment was due to internal translation initiating at AUG nucleotides 637–639 (amino acid M213, predicted molecular weight 18.53 kDa), which are highly conserved in vertebrates, including mammals, birds, and zebrafish (Additional file 2: Figure S1). Indeed, the internal translation hypothesis was recently elegantly confirmed by Smyth and Shaw using multiple approaches [13]. Our experiments provide additional data that support these key conclusions. We generated RNA of the Cx43 coding region *in vitro* and transfected Cx43-negative HeLa cells directly with this RNA. Direct transfection of Cx43 RNA avoids possible nuclear modifications such as splicing or alternative cryptic promoter usage. We observed a strong expression of Cx43 protein with nearly equal amounts of the 20-kDa immunoreactive band (Figure 1c), consistent with previous observations [13]. Smyth and Shaw have already ruled out protein degradation as a source of GJA1-20k by various means, such as by mutating the AUG start codon of full-length Cx43, which caused an even higher level of the alternative isoforms (including the 20-kDa fragment) in the absence of any full-length Cx43 (GJA1-43k) [13].

Smyth and Shaw [13] suggested that the internal translation was cap-independent, possibly due to the presence of one or more internal ribosome entry site (IRES) sequences that can mediate direct ribosomal entry and translation. Indeed, Cx43 has also been reported to contain an IRES sequence in the 5′UTR regulating full-length Cx43 translation [17]. However, various models of IRES translation exist and the mechanisms for eukaryotic genes remain controversial [18]. Three of our approaches suggest a slightly different explanation from that of Smyth and Shaw, involving cap-dependent ribosomal scanning or active upstream translation as a requirement to allow internal translation of *GJA1*. We transfected HeLa cells with RNA lacking the 5′-methyl-guanosine cap modification, m(7)G, which is required for eIF4E binding and standard cap-dependent mRNA translation [19]. Internal translation via a *bona fide* IRES sequence does not require cap-dependent translation and mRNA capping. As expected, we did not detect any cap-independent translation of full-length Cx43 protein when transfecting with RNA lacking the m(7)G cap. Unexpectedly, neither did we detect any cap-independent internally translated 20-kDa fragment (Figure 1c). However, this may have partially been due to RNA degradation of our RNA (since, by Q-PCR, we observe an approximate 40% reduction using uncapped versus capped RNA, Additional file 3: Figure S2C). To ensure that our observation was not due to degraded mRNA, or indeed due to uncapped RNA being stored in vesicles unable to initiate translation as reported after liposomal mRNA transfection [20], we used two alternative approaches to validate our hypothesis.

Firstly, we generated a Cx43 frameshift (FS) mutation that prevents the generation of full-length Cx43. For this approach, we took advantage of a naturally existing EcoNI site located only 30 base pairs after the full-length Cx43 AUG start codon. By cutting with EcoNI, removing the base pair overhangs using mung bean nuclease, followed by blunt-end ligation, we introduced a single base pair deletion to cause a FS mutation in Cx43 (see Additional file 1: Methods and Additional file 2: Figure S1). This FS-GJA1 mutation leads to a change in the amino acid (aa) sequence from aa12 onwards, followed by seven premature stop codons (between aa99 and aa181) before the putative 20-kDa AUG at aaM213 (see Additional file 2: Figure S1). We transfected Cx43 containing the FS mutation into Cx43-negative HeLa cells. Full-length GJA1-43k was not detectable (as expected). Furthermore, only a small amount of GJA1-20k was observed (again only from capped mRNA), and only after very long exposure of the blot (Figure 1c). In this respect, Smyth and Shaw performed a similar experiment by introducing three consecutive stop codons (at aa177–179) close to the 20-kDa M213 initiation site [13]. Again, significantly less GJA1-20k is generated compared to wild-type Cx43. Our FS-stop construct appears to inhibit GJA1-20k expression more efficiently however. This may be due to earlier termination (aa99) of Cx43 translation, although this remains speculative since the mechanism internal translation initiation remains unknown. Indeed, internal translation, particularly of mammalian genes, is complex, varied, and poorly elucidated [18], and therefore these putative novel observations will require extensive further studies.

Secondly, to further validate our findings that internal translation is dependent on a functional cap, ribosomal scanning or GJA1-43k translation, we inserted a highly stable hairpin (HP7) into the 5′UTR (ΔG = −61 kcal/mol; see Additional file 1: Methods and Additional file 2: Figure S1), previously described to efficiently prevent ribosomal scanning and cap-dependent translation [21,22]. Additionally, to generate a control readout of cap-independent translation within the same transcript, we inserted (downstream of Cx43) both the encephalomyocarditis virus (EMCV) IRES sequence plus an enhanced green fluorescent protein (EGFP) reporter. We generated capped RNA and transfected HeLa cells as before. As seen in Figure 1d, the HP7 hairpin efficiently blocks both GJA1-43k and -20k translation. Cap-independent EGFP translation from a viral IRES sequence—present in the same RNA transcript—is maintained however, as visualized in the same blot when including anti-GFP-HRP (Figure 2d).

Taken together, these results (using no cap, a FS mutation, or a stable hairpin blocking ribosomal scanning but not viral IRES activity), strongly suggest that unlike typical IRES sequences (e.g., EMCV), internal GJA1 translation is not mediated through a *bona fide* IRES sequence alone, and the presence of an intact classical 5′ methylated cap structure appears to be required. Furthermore, in addition to capped RNA, upstream active translation or ribosomal scanning (in a situation when M1 AUG is deleted) is required for efficient internal translation. We also conclude that this result is unlikely to be due to leaky ribosomal scanning of the M1 start codon, as the FS mutation in that setting would not be expected to have reduced GJA1-20k expression. These observations provide novel alternative insight into the various emerging concepts of mRNA translation that require further investigation [18].

### Translational regulation

We tested various pathways known to regulate mRNA translation. Through retroviral infections, we generated HeLa cell clones stably overexpressing chimeric Cx43-GFP (Figure 2a). In this system, we observed an increased GJA1-20k expression (20 kDa + 27 kDa GFP = 47 kDa) when inhibiting the mTOR pathway with rapamycin (Figure 2a), consistent with the report of Smyth and Shaw who used the mTOR inhibitor PP242 [13]. Moreover, inhibition of the Mnk1/2 kinases (MAP kinase-interacting serine/threonine-protein kinases 1/2) with the drug CGP 57380 also increased GJA1-20k expression, which was additive in combination with mTOR inhibition (Figure 2a). We also observed a strong induction of endogenous GJA1-20k in some cell lines, such as the breast cancer cell line MDA-MB-231, after Mnk1/2 inhibition by CGP 57380 (Figure 2b). Many tumours and cell lines have activated Mnk1/2 signalling as well as cytoplasmic Cx43 expression, and it will be of interest to explore whether inhibition of Mnk1/2 in a cancer setting induces GJA1-20k expression, thereby enhancing the membrane targeting of full-length Cx43, as already indicated after mTOR inhibition by Smyth and Shaw [13]. Increased membrane targeting may also explain why we see some increase in the stability of the full-length Cx43 protein, although we cannot exclude that Mnk1/2 and mTOR also regulate GJA1-43k expression directly and independently of GJA1-20k. Finally, as the EGFR pathway is frequently activated in cancers and can activate both mTOR and Mnk1, we treated cells with the EGFR inhibitor erlotinib. There was a modest increase after overnight treatment with 10 μM erlotinib (although a lower dose of 1 μM did not seem to be effective) (Figure 2a), indicating that the EGFR pathway and the linked PI3K/Akt pathway [13], both upstream of Mnk1/2, are important for the translational control of Cx43. In summary, these data demonstrate that several signalling pathways, including EGFR, Mnk1 (downstream of EGFR), and mTOR, can regulate the translation of GJA1-20k.

We also tested whether inhibition of Mnk1/2 regulated GJA1-20k expression in primary cells. Indeed there was a clear and significant increase in the amount of the 20-kDa fragment in both primary human fibroblasts and mouse embryonic fibroblasts treated with the Mnk1/2 inhibitor CGP 57380 (Figure 2c).

Mnk1/2 phosphorylates the translation initiation factor eIF4E [23], which is also regulated by mTOR (via 4EBP1) and is a rate-limiting factor for cap-dependent protein translation [24]. Mnk1/2 inhibition by CGP 57338 or through Mnk1/2 KO [23] does not affect total eIF4E expression as we [25] and others have consistently reported. In our study we also confirmed the previously reported complete block of eIF4E phosphorylation under these conditions (Figure 2b-d). How phospho-eIF4E regulates translation however, is controversial and still not well elucidated, but our study provides a useful new model to solve some of the outstanding questions. Accordingly, we are currently investigating whether eIF4E, an oncoprotein often overexpressed in cancer, is directly involved in the control of internal Cx43 translation. Cx43 does not seem to be particularly sensitive to eIF4E (nor mTOR nor Mnk1/2) as knocking down the cap-binding protein eIF4E (moderately) by siRNA does not strongly affect its expression levels although a modest reduction of GJA1-43k, and particularly of GJA1-20k, is observed (Additional file 3: Figure S2D). This result further supports the idea that GJA1-20k translation is cap-dependent, although the exact mechanism remains to be elucidated, as discussed above.

Finally, to conclusively link Mnk1/2 with Cx43 translation and to exclude potential nonspecific effects of the kinase inhibitor CGP 57380, we obtained mouse embryonic fibroblasts from Mnk1/2 knockout mice [23]. As with CGP 57380 treated cells, we observed an increase in GJA1-20k expression in knockout cells compared with wild-type cells (Figure 2d), conclusively demonstrating that this pathway regulates the ratio between full-length Cx43 and the 20-kDa isoform.

In conclusion, we show that GJA1-20k (corresponding to the Cx43 C-terminus) is present in a wide variety of cell lines as well as in primary human cells, and is produced through internal translation in a manner that is dependent on m7G-capping, ribosomal scanning and (at least to some extent) translation of full length Cx43. We show using inhibitors and cells from knockout mice that the Mnk1/2 kinase pathway, responsible for the phosphorylation of the translation initiation factor eIF4E, also regulates internal translation of the Cx43 transcript. It should be noted that our drug treatments do not lead to a reduction in full-length Cx43 due to reduced cap-dependent translation *per se*, as translation of only some transcripts are sensitive to these pathways. Finally, our RNA transfection studies suggest that there is no *bona fide* IRES element acting “independently”, since upstream translation, or scanning (when mutating the GJA1 M1 start codon [13]), appears to significantly aid internal translation. The exact underlying mechanism will be a fascinating future research topic. Further investigation into the regulation of the GJA1-43k/-20k ratio *in vitro* and *in vivo*, together with the elucidation of the exact functional consequences of this fragment, is warranted. This research will broaden our understanding of the gap junction-dependent and -independent functions of GJA1/Cx43 and the intricate mechanisms that allow protein diversity from a single mRNA transcript.

## Abbreviations

Aa: Amino acid; FS: Frameshift; IRES: Internal ribosome entry site; EMCV: Encephalomyocarditis virus; EGFP: Enhanced green fluorescent protein.

## Competing interests

The authors declare that they have no competing interests.

## Authors’ contributions

TA coordinated the project, designed the experiments and wrote the manuscript. CS, MS, CP, and TA performed the experiments. TA, CS, SRC and MS analysed the data. All authors read and approved the manuscript.

## Supplementary Material

Additional file 1 Supplementary Methods. Click here for file

Additional file 2: Figure S1 Translation of the GJA1 transcript. Diagram illustrating the Cx43 mRNA encoded from *GJA1*. The frameshift (FS) mutation at amino acid 12 leads to seven putative stop codons (at aa99, 106, 127, 132, 145, 171, 181) before the translation initiation site of GJA1-20k at aaM213. Other putative start codons are also indicated. The sequence surrounding the GJA1-20k start codon is indicated in various species down to zebrafish, indicating a highly conserved region. The protein products of GJA1-43k (with a four-transmembrane topology) and GJA1-20k are indicated. GJA1-20k is, relative to full-length Cx43 topology, thought to initiate inside the fourth transmembrane region. The location of the HP7 hairpin to block ribosomal scanning, a putative area for internal IRES sequence, and the C-terminal antibody binding region are indicated. Click here for file

Additional file 3: Figure S2 Additional evidence for the concomitant regulation of GJA1-43k and GJA1-20k. (A) Primary human keratinocytes cultured in low calcium and in high calcium to stimulate differentiation (as measured by the differentiation marker involucrin) show concomitant regulation of Cx43 (GJA1-43k) and GJA1-20k. (B) Knockdown of Cx43 with esiRNA, or by microRNA-1 or −206, shows the concomitant regulation of full-length Cx43 GJA1-43k and the GJA1-20k isoform in lung BT-549 breast cancer cells. (C) Q-PCR indicates a moderate reduction in GJA1 transcript abundance of around 40% comparing of uncapped versus capped RNA. (D) siRNA-mediated knockdown of eIF4E leads to a moderate reduction in GJA1-43k and GJA1-20k levels in A-549. Click here for file
