# Supplementary material for: Internal translation of the connexin 43 transcript
Source: Cell Commun Signal. 2014 May 8;12:31. doi: 10.1186/1478-811X-12-31 (PMC4108066; doi:10.1186/1478-811X-12-31)
Supplement: Additional file 1 — Supplementary Methods. [file 1478-811X-12-31-S1.pdf]

Supplementary Methods

Cell culture and treatments

Primary human keratinocytes and fibroblasts were obtained and cultured as previously described [1]. Wild-type and Mnk1/2 knockout mouse embryonic fibroblasts (MEFs) [2] were a gift from the Nahum Sonenberg laboratory (McGill University, Canada), and were cultured in DMEM with 10% FBS and 1% penicillin/streptomycin. All other cell lines were obtained from ATCC and cultured as recommended in DMEM with 10% FBS and 1% penicillin/streptomycin. Cells were treated overnight (unless indicated otherwise in the manuscript) with 40 µM CGP 57380 (Tocris Bioscience) or 100 nM Rapamycin (Tocris Bioscience).

RNA interference and microRNA studies.

Mission endoribonuclease-prepared siRNA (esiRNA) control and esiRNA targeting the entire *GJA1* coding region (obtained from Sigma) were transfected with RNAiMax according to the recommended protocol (Life Technologies) using 1000ng esiRNA per well in 6-well plates. Targeting eIF4E was performed with a proven standard Flexitube siRNA from Qiagen (#SI00300125). Hsa-miR-1, has-miR-206 and –ve control miR-mimick were obtained from Ambion (#4464066, Life Technologies) and transfected with RNAiMax to a final concentration of 25 nM. Cells were harvested 48 hours post-transfection.

Western blotting and antibodies

Cells were lysed in RIPA buffer (Santa Cruz) and western blotting was performed as previously described [3]. The antibodies used were rabbit polyclonal anti-Cx43 directed against the C-terminal (abcam #ab11-370, 1:10000, or Sigma Aldrich

#C6219, 1:10000), mouse monoclonal anti–a-tubulin (abcam #ab7291, 1:10000), anti-GFP (abcam #ab1218, 1:5000), HRP-conjugated anti-GFP (MACS Milteny Biotech #130-091-833, 1:5000), anti-eIF4E (Cell Signalling, #9742S, 1:1000), anti-phospho-eIF4E-Ser209 (Novus Biochemicals #NB100-79928, 1:10000) and anti-vinculin (Sigma Aldrich #V4505, 1:1000).

Cloning and mutagenesis

A Cx43 frameshift mutation was introduced as follows. Wild-type Cx43 (in pBluescript) was digested with EcoNI (single site in Cx43 at base pair 30) and the single linearized plasmid was isolated from an agarose gel by a standard gel isolation procedure. The overlapping 5' and 3' base pairs after cutting with EcoNI were removed by incubating with mung bean nuclease following the recommended procedure, and the blunted ends were re-ligated and transformed into competent *E. coli*. Colonies were isolated, and successfully mutated plasmids were identified by EcoNI/NotI double digest. The exact mutation was verified by sequencing. IRES-GFP were isolated from pIRES2-GFP (Clontech) by BamHI + NotI digest and inserted downstream of Cx43 in pBluescript. A $\Delta G = -61$ kcal/mol stable hairpin HP7, GGGGCGCGTGGTGGCGGCTGCAGCCGCCACCACGCGCCCC, previously described to inhibit ribosomal scanning and cap-dependent translation [4, 5], was inserted into the XhoI / HindIII sites of pBluescript-Cx43-IRES-GFP by oligocloning (Life Technologies).

***In vitro* transcription of Cx43 RNA**

Cx43 and Cx43-IRES-GFP in pBluescript containing the coding sequence with a 5' T7 sequence were linearized, gel-purified and subjected to *in vitro* RNA transcription

using the T7 mScript™ Standard mRNA Production System (C-MSC11610; CellScript) following the manufacturer's recommendations. Isolated RNA was quantified using a NanoDrop 2000 (Thermo Scientific).

Transfections and retroviral production and infections

Cells in 6-well plates were transfected with 2 µg RNA using RNAiMax (Life Technologies) following the manufacturer's recommended standard procedure. Cells were harvested 8 or 24 hours post-transfection. Retrovirus containing Cx43-GFP was produced and used to infect HeLa cells as previously described [1]. Stable clones were obtained by selection of individual clones positive for Cx43-GFP under fluorescence microscopy.

Quantitative real time PCR

HeLa cells were cotransfected with RNA encoding for Cx43 (with or without m7G-cap structure) and RNA encoding for Firefly Luciferase (FLuc), which was used as a transfection control. Total RNA from cells was isolated using the RNeasy Plus Mini Kit (Qiagen) following the manufacturer’s instructions. Complementary DNA (cDNA) was obtained after reverse transcripition of total RNA (1 µg) using the Maxima First Strand cDNA Synthesis kit (ThermoScientific) with Random Hexamers. SYBR Green Real Time PCR was performed using 10 µl of Sybr Select Master Mix (Life Technologies), 5 µl of cDNA (5ng/µl) and 0.4 µl of each forward and reverse primer at 10 µM. The primers used were: GJA1, forward 5’-ATGAGCAGTCTGCCTTTCGT-3’, reverse TCTGCTTCAAGTGCATGTCC ; FLuc, forward 5’-ACAGATGCACATATCGAGGTG-3’, reverse 5’-GATTTGTATTCAGCCCATATCG-3’; β-actin, forward 5’-

GCAAAGACCTGTACGCCAAC-3’, reverse 5’-AGTACTTGCGCTCAGGAGGA-3’.

References:

1. Aasen T, Belmonte JC: **Isolation and cultivation of human keratinocytes from skin or plucked hair for the generation of induced pluripotent stem cells**. *Nat Protoc* 2010, **5**(2):371-382.
2. Ueda T, Watanabe-Fukunaga R, Fukuyama H, Nagata S, Fukunaga R: **Mnk2 and Mnk1 are essential for constitutive and inducible phosphorylation of eukaryotic initiation factor 4E but not for cell growth or development**. *Mol Cell Biol* 2004, **24**(15):6539-6549.
3. Macdonald AI, Sun P, Hernandez-Lopez H, Aasen T, Hodgins MB, Edward M, Roberts S, Massimi P, Thomas M, Banks L *et al*: **A functional interaction between the MAGUK protein hDlg and the gap junction protein Connexin 43 in cervical tumour cells**. *Biochem J* 2012.
4. Kozak M: **Circumstances and mechanisms of inhibition of translation by secondary structure in eucaryotic mRNAs**. *Mol Cell Biol* 1989, **9**(11):5134-5142.
5. Kronstad LM, Brulois KF, Jung JU, Glaunsinger BA: **Dual short upstream open reading frames control translation of a herpesviral polycistronic mRNA**. *PLoS pathogens* 2013, **9**(1):e1003156.
