# Supplementary figures and images for: Internal translation of the connexin 43 transcript
Source: Cell Commun Signal. 2014 May 8;12:31. doi: 10.1186/1478-811X-12-31 (PMC4108066; doi:10.1186/1478-811X-12-31)

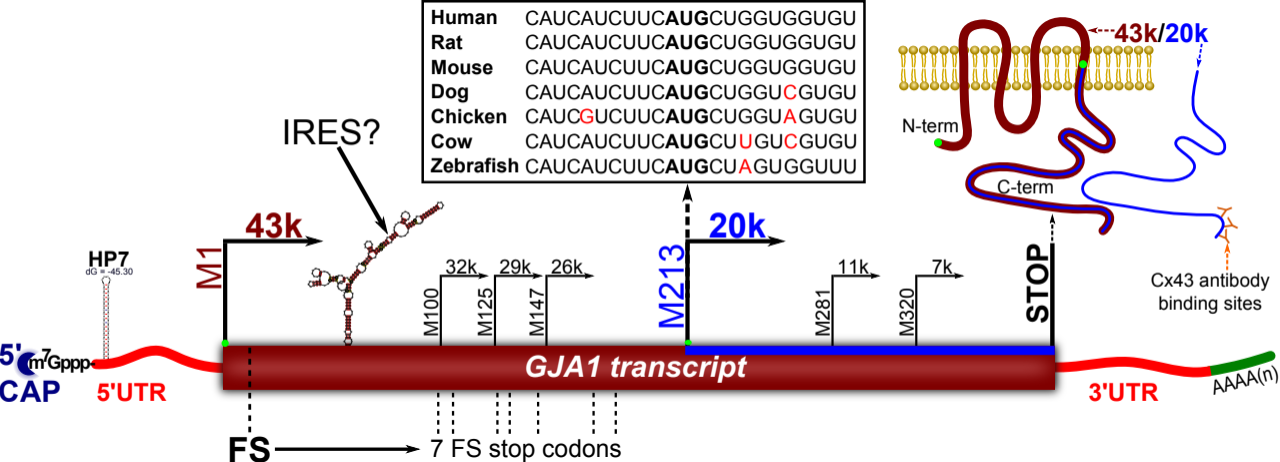

Supplement: Additional file 2: Figure S1 — Translation of the GJA1 transcript. Diagram illustrating the Cx43 mRNA encoded from GJA1. The frameshift (FS) mutation at amino acid 12 leads to seven putative stop codons (at aa99, 106, 127, 132, 145, 171, 181) before the translation initiation site of GJA1-20k at aaM213. Other putative start codons are also indicated. The sequence surrounding the GJA1-20k start codon is indicated in various species down to zebrafish, indicating a highly conserved region. The protein products of GJA1-43k (with a four-transmembrane topology) and GJA1-20k are indicated. GJA1-20k is, relative to full-length Cx43 topology, thought to initiate inside the fourth transmembrane region. The location of the HP7 hairpin to block ribosomal scanning, a putative area for internal IRES sequence, and the C-terminal antibody binding region are indicated. [file 1478-811X-12-31-S2.pdf]

a)

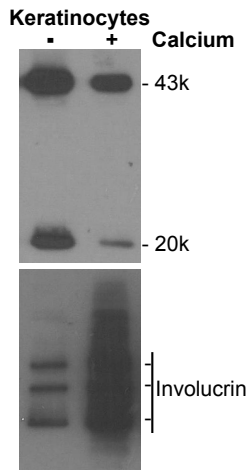

b)

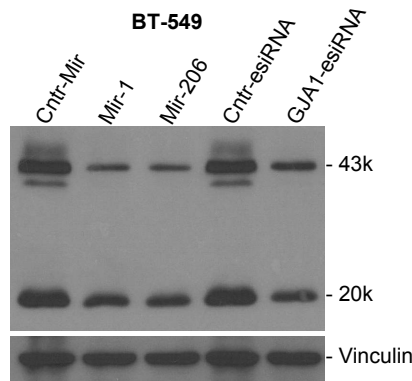

c)

### QPCR of Cx43 transcript abundance

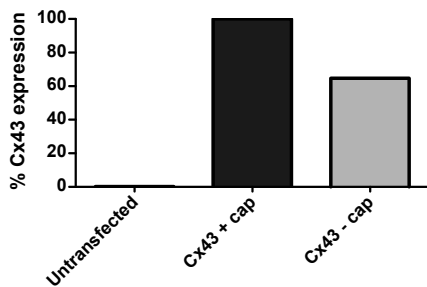

d)

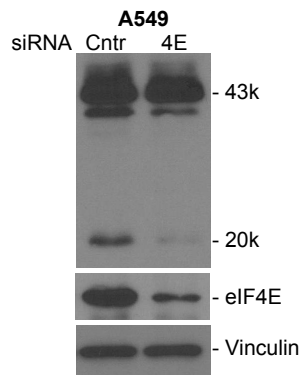

Supplement: Additional file 3: Figure S2 — Additional evidence for the concomitant regulation of GJA1-43k and GJA1-20k. (A) Primary human keratinocytes cultured in low calcium and in high calcium to stimulate differentiation (as measured by the differentiation marker involucrin) show concomitant regulation of Cx43 (GJA1-43k) and GJA1-20k. (B) Knockdown of Cx43 with esiRNA, or by microRNA-1 or −206, shows the concomitant regulation of full-length Cx43 GJA1-43k and the GJA1-20k isoform in lung BT-549 breast cancer cells. (C) Q-PCR indicates a moderate reduction in GJA1 transcript abundance of around 40% comparing of uncapped versus capped RNA. (D) siRNA-mediated knockdown of eIF4E leads to a moderate reduction in GJA1-43k and GJA1-20k levels in A-549. [file 1478-811X-12-31-S3.pdf]
